# From mouse to man: safety, immunogenicity and efficacy of a candidate leishmaniasis vaccine LEISH-F3+GLA-SE

**DOI:** 10.1038/cti.2015.6

**Published:** 2015-04-10

**Authors:** Rhea N Coler, Malcolm S Duthie, Kimberly A Hofmeyer, Jeffery Guderian, Lakshmi Jayashankar, Julie Vergara, Tom Rolf, Ayesha Misquith, John D Laurance, Vanitha S Raman, H Remy Bailor, Natasha Dubois Cauwelaert, Steven J Reed, Aarthy Vallur, Michelle Favila, Mark T Orr, Jill Ashman, Prakash Ghosh, Dinesh Mondal, Steven G Reed

**Affiliations:** 1 Infectious Disease Research Institute, Seattle, WA, USA; 2 International Center for Diarrhoeal Diseases Research, Centre for Nutrition and Food Security, Parasitology Laboratory, Dhaka, Bangladesh

## Abstract

Key antigens of *Leishmania* species identified in the context of host responses in *Leishmania*-exposed individuals from disease-endemic areas were prioritized for the development of a subunit vaccine against visceral leishmaniasis (VL), the most deadly form of leishmaniasis. Two *Leishmania* proteins—nucleoside hydrolase and a sterol 24-c-methyltransferase, each of which are protective in animal models of VL when properly adjuvanted— were produced as a single recombinant fusion protein NS (LEISH-F3) for ease of antigen production and broad coverage of a heterogeneous major histocompatibility complex population. When formulated with glucopyranosyl lipid A-stable oil-in-water nanoemulsion (GLA-SE), a Toll-like receptor 4 T_H_1 (T helper 1) promoting nanoemulsion adjuvant, the LEISH-F3 polyprotein induced potent protection against both *L. donovani* and *L. infantum* in mice, measured as significant reductions in liver parasite burdens. A robust immune response to each component of the vaccine with polyfunctional CD4 T_H_1 cell responses characterized by production of antigen-specific interferon-γ, tumor necrosis factor and interleukin-2 (IL-2), and low levels of IL-5 and IL-10 was induced in immunized mice. We also demonstrate that CD4 T cells, but not CD8 T cells, are sufficient for protection against *L. donovani* infection in immunized mice. Based on the sum of preclinical data, we prepared GMP materials and performed a phase 1 clinical study with LEISH-F3+GLA-SE in healthy, uninfected adults in the United States. The vaccine candidate was shown to be safe and induced a strong antigen-specific immune response, as evidenced by cytokine and immunoglobulin subclass data. These data provide a strong rationale for additional trials in *Leishmania*-endemic countries in populations vulnerable to VL.

Leishmaniasis, a sandfly-transmitted series of poverty-related diseases caused by infection with *Leishmania* species parasites, threatens about 350 million people in 98 countries around the world. It has a wide range of clinical presentations that are dependent on the infecting strain and may cause cutaneous, mucosal or visceral leishmaniasis (CL, MCL or VL). VL, also known as kala-azar, is the most severe form of leishmaniasis, and is typically fatal if left untreated.^1^ VL in South Asia and East Africa are caused by infection with *Leishmania donovani*, whereas in the Mediterranean, the Middle East and Latin America, *L. infantum* is the causative agent.^2^ An estimated 200 000 to 400 000 VL cases are reported annually.^3^ Although various chemotherapies for VL exist (for example, antimonials, miltefosine, paramomycin and amprotericin B), they can be toxic, and as a result of repeated, long-term use, drug-resistant parasites are becoming more frequent in endemic areas. A VL elimination campaign has been initiated in India, Bangladesh and Nepal, which will focus on vector control, early diagnosis and drug treatment strategies,^4, 5, 6^ but it is likely that a vaccine will be needed for successful elimination.^3^

The ability of *Leishmania* infection to generate life-long immunity offers promise for the development of a vaccine for VL.^7^ Computer simulation indicates that vaccination would be a cost-effective control measure,^8^ capable of providing long-term protection against disease as well as reducing anthroponotic transmission and persistent reservoirs of parasites. A limitation to an effective vaccine for leishmaniasis is the absence of information on immunological correlates of natural and vaccine-mediated protection in humans. Previous studies have shown the requirement of parasite-specific T_H_1 (T helper 1) responses, characterized by the production of interferon-γ (IFNγ), interleukin-2 (IL-2) and tumor necrosis factor (TNF) by polyfunctional CD4^+^ T cells, and the balance between immunoregulatory mechanisms of proinflammatory IFNγ/TNF-α and regulatory IL-10 cytokines.^9, 10, 11, 12, 13, 14^

The use of drugs plus whole parasites or crude antigens adsorbed with alum plus bacillus Calmette–Guerin or appropriate adjuvants has demonstrated that vaccination against leishmaniasis is possible.^2^ However, difficulty in standardizing formulation and dosing of live vaccines or crude preparations makes these approaches impractical and inconsistent. A selection of defined antigens including LEISH-111f developed by our group has been studied as vaccine antigen candidates for leishmaniasis in animal models and clinical studies. Using a combination of reverse vaccinology and bioinformatics approaches, we previously identified 43 *Leishmania* proteins recognized by antibodies in the serum of Sudanese VL patients as potential vaccine antigen candidates.^15^ Our selection criteria for vaccine development included sequence conservation across *Leishmania* spp., a lack of sequence identity with human genes and protective efficacy in animal models. Two *Leishmania* proteins from our screen that met these criteria were nucleoside hydrolase (NH) and sterol 24-c-methyltransferase (SMT), which we have combined into the fusion antigen construct NS (hereafter referred to as LEISH-F3). NH is a 35 kDa glycoprotein and a main component of the highly antigenic fucose–mannose ligand complex of *L. donovani*, which induces strong immune responses and protects mice against experimental *L. donovani*,^16^ *L. chagasi* and *L. mexicana* infections.^17^ When formulated with QuilA saponin, NH also protects dogs against VL.^18^ *L. infantum* SMT is a 40 kDa enzyme with an amino-acid sequence that is highly conserved across many *Leishmania* species, but is absent in mammals.^19^ When appropriately formulated with a Toll-like receptor 4 (TLR-4) adjuvant, SMT provides protection against both *L. infantum* and *L. major* experimental infections.^15, 19, 20^

In general, recombinant proteins are poorly immunogenic and require the addition of an adjuvant to elicit adaptive immune responses. To address this issue, we developed a synthetic TLR-4 ligand, glucopyranosyl lipid A (GLA).^21, 22^ When formulated in a stable oil-in-water nanoemulsion (SE), the GLA-SE adjuvant system induces antigen-specific T_H_1 immune responses that are associated with efficacy in several animal models of infection, including tuberculosis, malaria and influenza.^23, 24, 25, 26, 27, 28, 29, 30, 31, 32, 33, 34, 35^ Completed and ongoing clinical trials have shown our GLA-based formulations to be both safe and immunogenic in vaccine strategies for infectious diseases, allergy and cancer.^36, 37^

Here we report recognition of NH and SMT in the sera of *Leishmania*-infected individuals from disease-endemic areas of Bangladesh and the development of a recombinant fusion antigen containing these two proteins, LEISH-F3. We characterized the immune responses induced by immunization of LEISH-F3 formulated with GLA-SE (LEISH-F3+GLA-SE), and demonstrate in mouse models of VL that prophylactic immunization confers protection against both causative strains of VL, *L. donovani* and *L. infantum*. We also report results for a first-in-human study that evaluated LEISH-F3+GLA-SE in healthy adults from a non-endemic region. As predicted by our preclinical studies, immunization with LEISH-F3+GLA-SE in a human clinical trial proved to be safe and highly immunogenic; in immunized subjects, we observe a substantial anti-LEISH-F3 serum antibody response and production of key protective cytokines by LEISH-F3-recalled peripheral blood mononuclear cells (PBMCs), which may be potent contributors to vaccine efficacy.

## RESULTS

### Immune recognition of NH and SMT by individuals from a VL-endemic area in Bangladesh

**Figure 1:**
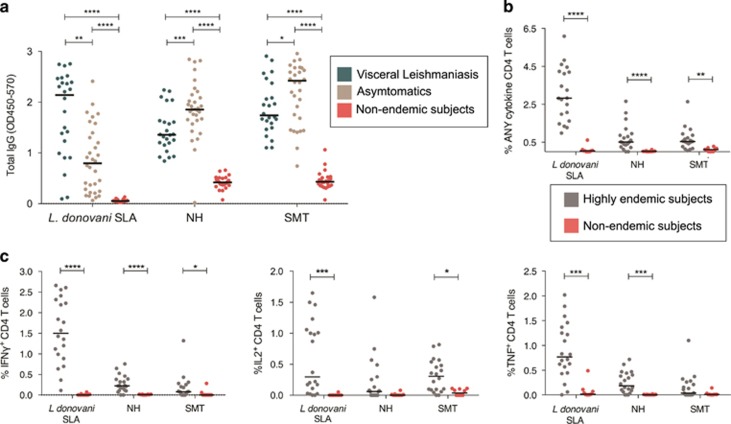
Recognition of NH and SMT antigens in patients from a *L. donovani* endemic area in Bangladesh. (**a**) Total IgG against *L. donovani* SLA, NH and SMT are shown. Antibodies against the antigens were measured by ELISA in sera (used at 1:400 dilution) in VL individuals (*n*=25), asymptomatic individuals (*n*=32) and non-endemic controls (*n*=24). (**b**) PMBCs were recalled with 10 μg ml^−1^ *L. donovani* SLA, NH or SMT and analyzed for CD4 T cells production of indicated cytokines by flow cytometry. (**a** and **b**) Black bars indicate median OD for each group. Statistical significance indicated is vs the non-endemic normal group; statistic was calculated by Kolmogorov–Smirnov *t*-test. ****P<0.0001.

To validate the use of the NH and SMT proteins as potential vaccine candidates, we first evaluated whether they were recognized by antibodies in sera from *Leishmania*-exposed individuals from a disease-endemic area in Bangladesh (Figure 1). Endemic area residents were defined as either having active VL or as asymptomatics, and compared with healthy individuals from a non-endemic area (USA). To determine if sera from *Leishmania*-exposed individuals recognize NH or SMT, we performed enzyme-linked immunosorbent assays (ELISAs) for total immunoglobulin G (IgG) (Figure 1a) and compared recognition of our antigens with recognition of *L. donovani* soluble lysate antigen (SLA). Both VL and asymptomatic individuals had significantly higher anti-NH, anti-SMT and anti-*L. donovani* SLA serum IgG compared with non-endemic individuals (Figure 1a). Asymptomatic individuals had lower anti-*L. donovani* SLA compared those with VL, but had higher anti-NH and anti-SMT serum IgG compared with VL (Figure 1a).

The antigenicity of NH and SMT was then evaluated by *ex vivo* stimulation of PBMCs collected from asymptomatic individuals. Cells were used to characterize CD4^+^ T cells producing IFNγ, TNF and IL-2, following stimulation with *L. donovani* SLA, NH and SMT. PBMCs from non-endemic US subjects were used as negative controls. As expected, CD4^+^ T-cell IFNγ, TNF and IL-2 cytokine responses were significantly higher in the asymptomatic individuals from a VL hyperendemic region when recalled with the antigens as compared with the non-endemic healthy controls (Figure 1b).

### Generation and characterization of the LEISH-F3 fusion protein construct

**Figure 2:**
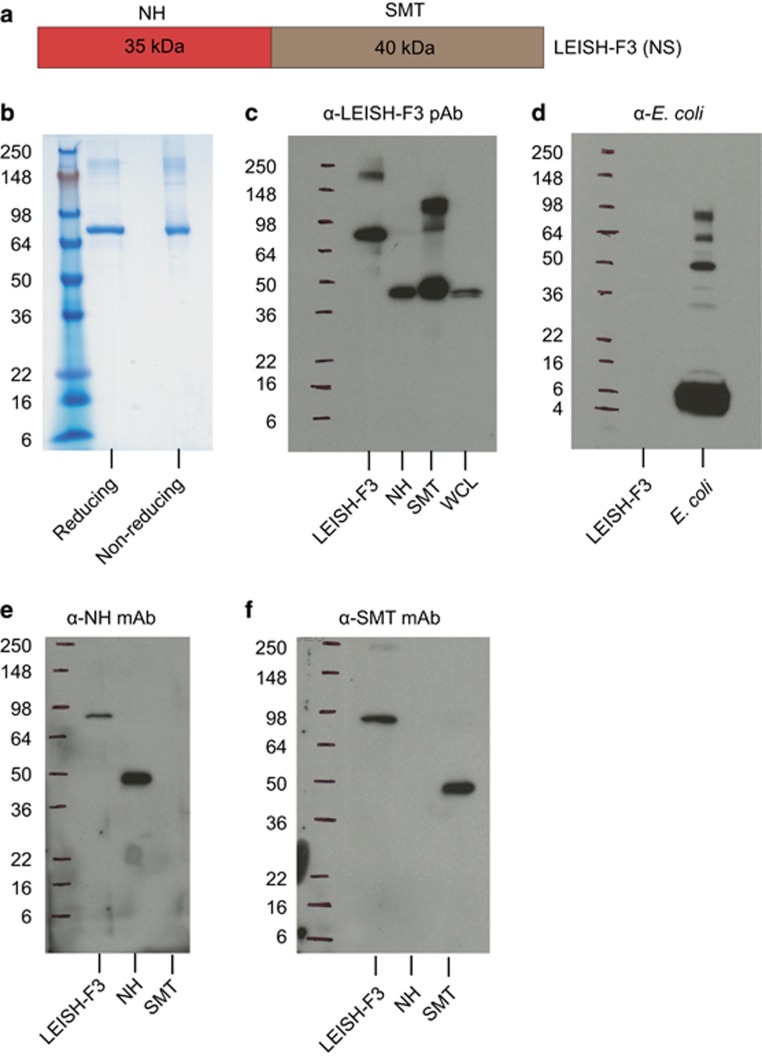
LEISH-F3 construct and characterization. (**a**) Schematic of LEISH-F3 (NS) fusion protein. (**b**–**e**) SDS-PAGE and immunoblot of LEISH-F3 (NS). (**b**) LEISH-F3 was run in reducing and nonreducing conditions on a 4–20% Tris-glycine gel. (**c**) Immunoblot of LEISH-F3 with rabbit polyclonal antibody to LEISH-F3; 100 ng each of LEISH-F3, NH and SMT; 27 μg of *L. donovani* whole-cell lysate (WCL). (**d**) Immunoblot of 2 μg of LEISH-F3 and HMS174 *E. coli* lysate with antibody to HMS174 *E. coli*. (**e**, **f**) Immunoblot of LEISH-F3 and its component antigens, NH and SMT, with mouse monoclonal antibody (mAb) against (**e**) NH or (**f**) SMT; 100 ng each of LEISH-F3, NH and SMT were loaded.

Having demonstrated IgG and cell-mediated responses against NH and SMT in *Leishmania*-infected individuals, as well as immunogenicity and protection against *L. donovani*, a straightforward approach was to link in tandem the open reading frames of both genes such that the final construct would result in the generation of a single recombinant antigen, NS/LEISH-F3, comprising both antigens for evaluation as a VL vaccine candidate (Figure 2a). The *L. donovani* sequence for NH and the *L. infantum* sequence for SMT were used for the LEISH-F3 construct design. Sodium dodecyl sulfate-polyacrylamide gel electrophoresis analysis of purified, scaled-up fermentation lots of LEISH-F3 revealed minor protein aggregation and a single major band at the expected molecular mass of 75 kDa. A similar band profile was observed for samples in reducing and nonreducing conditions, demonstrating the relative absence of protein aggregates (Figure 2b). The identity of the major band, relative absence of aggregates and large protein fragments were confirmed by immunoblotting with a mouse polyclonal serum raised against LEISH-F3 (Figure 2c). The anti-LEISH-F3 polyclonal antibody recognized multiple epitopes and therefore multiple forms of the proteins. As the higher-molecular-weight species is seen with the SMT protein, it suggests that this component of LEISH-F3 is responsible for the multimerization. Furthermore, the absence of bacterial by-products in the purified LEISH-F3 product was confirmed by immunoblotting with polyclonal antibody to *Escherichia coli* (Figure 2d). Finally, LEISH-F3 was recognized by monoclonal sera raised against a single epitope on each of the single components, confirming the presence of both NH and SMT within the fusion protein (Figure 2e). Based on high levels of homology in the sequence of the *NH* and *SMT* genes across additional *Leishmania* species (Table 1), it is reasonable to assume that these proteins are expressed and recognized during the infectious process of multiple *Leishmania* species.

### The LEISH-F3+GLA-SE vaccine is immunogenic and generates an antigen-specific T_H_1 immune response in a preclinical mouse model

**Figure 3:**
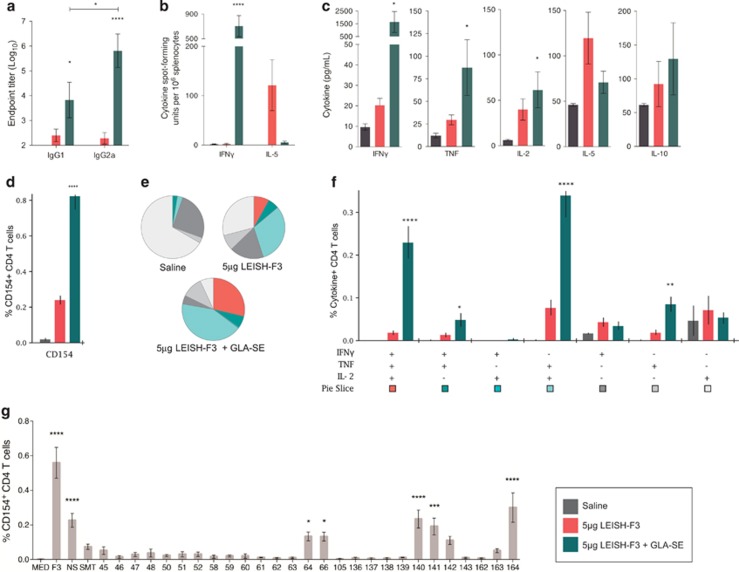
LEISH-F3+GLA-SE is immunogenic and generates an antigen-specific T_H_1 memory response. BALB/c mice were immunized with the indicated formulations three times, at 3-week intervals, for the analysis of LEISH-F3-specific immune responses. (**a**) Serum was collected 4 weeks after the final immunization and analyzed for LEISH-F3-specific antibodies by ELISA end-point titration (*n*=5). Statistics within isotypes (vs saline) and between isotypes by Sidack's multiple comparison test. *P<0.05 and ****P<0.0001. (**b**–**e**) LEISH-F3-specific immune responses were analyzed by recall of splenocytes with 10 μg ml^−1^ LEISH-F3 from mice 4 weeks after the final immunization. (**b**) ELISPOT assay of IFNγ and IL-5 (*n*=4); statistics by Dunnett's multiple comparison test within each cytokine vs saline. ****P<0.0001. (**c**) Cytokine bead array of cytokines in the supernatant of splenocytes recalled for 72 h (*n*=3). Statistics by Dunnett's multiple comparison test vs saline. *P<0.05. (**d**) Percentage of CD4 T cells expressing CD154 (*n*=5); statistics by Dunnett's multiple comparison test. *P<0.05 and ****P<0.0001. (**e** and **f**) Percentages of polyfunctional CD4 T cells expressing combinations of IFNγ, TNF and IL-2 (*n*=5). (**f**) Statistics by Dunnett's multiple comparison test within polyfunctional cytokine groups vs saline. *P<0.05, **P<0.01 and ****P<0.0001. (**g**) Major histocompatibility complex (MHC-II) epitope mapping of the LEISH-F3 fusion protein in LEISH-F3+GLA-SE-immunized mice. Statistics by Student's *t*-test vs media. *P<0.05 and **P<0.01.

To determine the immunogenic potential of NH, SMT and LEISH-F3 as vaccines, we formulated the single components and the fusion antigen with the synthetic nanoemulsion GLA-SE for immunization. Mice received three immunizations at 3-week intervals and antigen-specific antibody and T-cell responses were measured 4 weeks after the final immunization and compared with saline or LEISH-F3-alone controls (Supplementary Figure S1). Compared with control immunizations, LEISH-F3+GLA-SE generated higher anti-LEISH-F3 IgG1 and IgG2a serum antibodies; LEISH-F3+GLA-SE generated significantly higher titers of IgG2a than IgG1 (Figure 3a). A positive IgG2a:IgG1 ratio is indicative of an IFNγ-dependent isotype switch in IgG subclasses typical of a T_H_1-biased immune response.

To demonstrate formally that antigen-specific T_H_1 responses had been induced in immunized mice, splenocytes were recalled with LEISH-F3 protein *ex vivo.* We observed a T_H_1-skewed memory response with IFNγ, but not IL-5, producing cells readily detected in LEISH-F3+GLA-SE-immunized mice (Figure 3b and Supplementary Figure S1). The inverse was observed upon recall of cells from mice immunized with LEISH-F3 alone, indicating the importance of the GLA-SE adjuvant to subvert a T_H_2 response in favor of T_H_1 (Figure 3b). This was also reflected in the amount of total cytokines produced by LEISH-F3-recalled splenocytes isolated from LEISH-F3+GLA-SE-immunized mice, which had enhanced production of key T_H_1 cytokines IFNγ, TNF-α and IL-2 and low levels of the T_H_2/Treg cytokines IL-5 and IL-10 (Figure 3c).

We further characterized the immune response generated following our immunization regimen with LEISH-F3+GLA-SE by analyzing the LEISH-F3-specific T-cell memory response using multiparameter flow cytometry and intracellular cytokine staining. Although LEISH-F3-specific CD8 T cells responses were not observed, LEISH-F3+GLA-SE immunization generated LEISH-F3-specific CD4 T cells as indicated by the higher percentage of these cells expressing CD154 (a ligand expressed by recently activated CD4 T cells) following *ex vivo* recall with LEISH-F3 (Figure 3d). LEISH-F3-specific CD4 T cells from LEISH-F3+GLA-SE mice proved to be T_H_1 skewed and polyfunctional (Figures 3e and f). Mice that received the LEISH-F3+GLA-SE vaccine showed a significant increase in the proportion of LEISH-F3-specific CD4 T cells producing all three of the key T_H_1 cytokines (IFNγ, IL-2 and TNF; 0.23% of CD4 T cells) or some combination of two (0.40% of CD4 T cells) compared with the untreated control; a total of 0.63% of CD4 T cells from LEISH-F3+GLA-SE mice were polyfunctional in contrast to 0.11% of the LEISH-F3-alone immunization. Thus, immunization of mice with LEISH-F3+GLA-SE induces polyfunctional antigen-specific T_H_1 responses, which are classically associated with protection against experimental *Leishmania* infection.^38^

We also conducted CD4 T-cell epitope mapping of LEISH-F3- in the LEISH-F3+GLA-SE-immunized mice. A panel of 164 peptides, each 15-amino-acid long and overlapping by 11 residues, were organized into a two-dimensional matrix of 26 peptides pools, so that individual peptides were present in two independent pools. When a positive response was observed from a peptide in both peptide pools, it was further evaluated in follow-up splenocyte recalls to determine the actual individual peptide that induced positive responses.^39, 40^ We found that BALB/c mice had several CD4 epitopes for LEISH-F3 in both the NH and SMT portions of the fusion antigen (Figure 3g).

### Immunization with LEISH-F3+GLA-SE induces protection against experimental VL infection with both *L. donovani* and *L. infantum*

**Figure 4:**
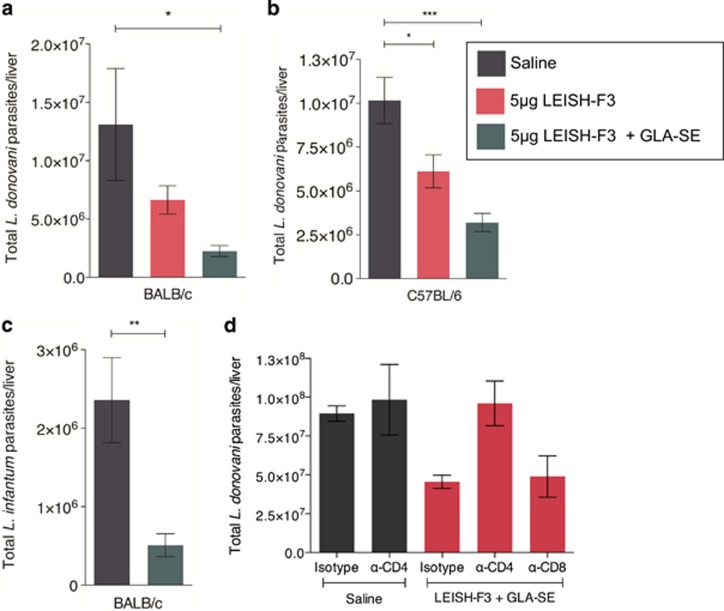
CD4 memory T cells generated by vaccination with LEISH-F3+GLA-SE protect against experimental VL infection. (**a** and **c**) BALB/c and (**b** and **d**) C57BL/6 mice were immunized with the indicated vaccines three times, at 3-week intervals. (**a**–**c**) Four weeks after the final immunization, mice were infected with (**a** and **b**) 1 × 10^6^  *L. donovani* or (**c**) 5 × 10^6^ *L. infantum* parasites. (**d**) Three weeks after the final immunization, mice received three intraperitoneal injections, on 3 consecutive days, of 0.5 mg of the indicated depleting antibody; 3 weeks later, mice were infected with 1 × 10^6^ *L. donovani* parasites intravenously. (**a**–**d**) Livers were harvested 3 weeks after infection and analyzed by reverse transcription-PCR (RT-PCR) to determine the total parasite burden. (**a** and **b**) Statistics by Tukey's multiple comparison test. *P<0.05 and ***P<0.005. (**c** and **d**) Statistics by unpaired *t*-test between indicated groups. *P<0.05, **P<0.01 and ****P<0.001.

To evaluate the protective potential of immunization with NH, SMT or LEISH-F3, each formulated with GLA-SE, BALB/c and C57BL/6 mice were immunized and experimentally infected with *L. donovani* or *L. infantum* 4 weeks after the final immunization. Control mice received saline injections, as our prior experience demonstrated that injection with GLA-SE alone in the same regimen does not change infection relative to treatment with saline (data not shown). Parasite burdens in livers were quantified 4 weeks after infection. A significant reduction of *L. donovani* parasites was observed in both BALB/c and C57BL/6 mice immunized with NH, SMT or LEISH-F3 formulated with GLA-SE compared with mice given the control preparations (Figures 4a and b and Supplementary Figure S1). Owing to the high cross-*Leishmania* species homology of NH and SMT (Table 1), we evaluated if immunization with LEISH-F3+GLA-SE could also reduce parasite burdens in mice infected with the other causative strain of VL, *L. infantum.* We found that the LEISH-F3+GLA-SE-immunized mice had significantly reduced *L. infantum* parasite burdens (Figure 4c). The importance of memory CD4 T cells generated by LEISH-F3+GLA-SE immunization in protection against *L. donovani* infection was examined by depleting either CD4 or CD8 T cells in mice subjected to the standard immunization regimen before infection with *L. donovani*. In LEISH-F3+GLA-SE-immunized mice, depletion of CD4 T cells, but not CD8 T cells, resulted in the loss of protection and parasite liver burdens comparable to the unprotected saline-immunized mice (Figure 4d).

### Clinical characterization of subjects vaccinated with LEISH-F3+GLA-SE

**Figure 5:**
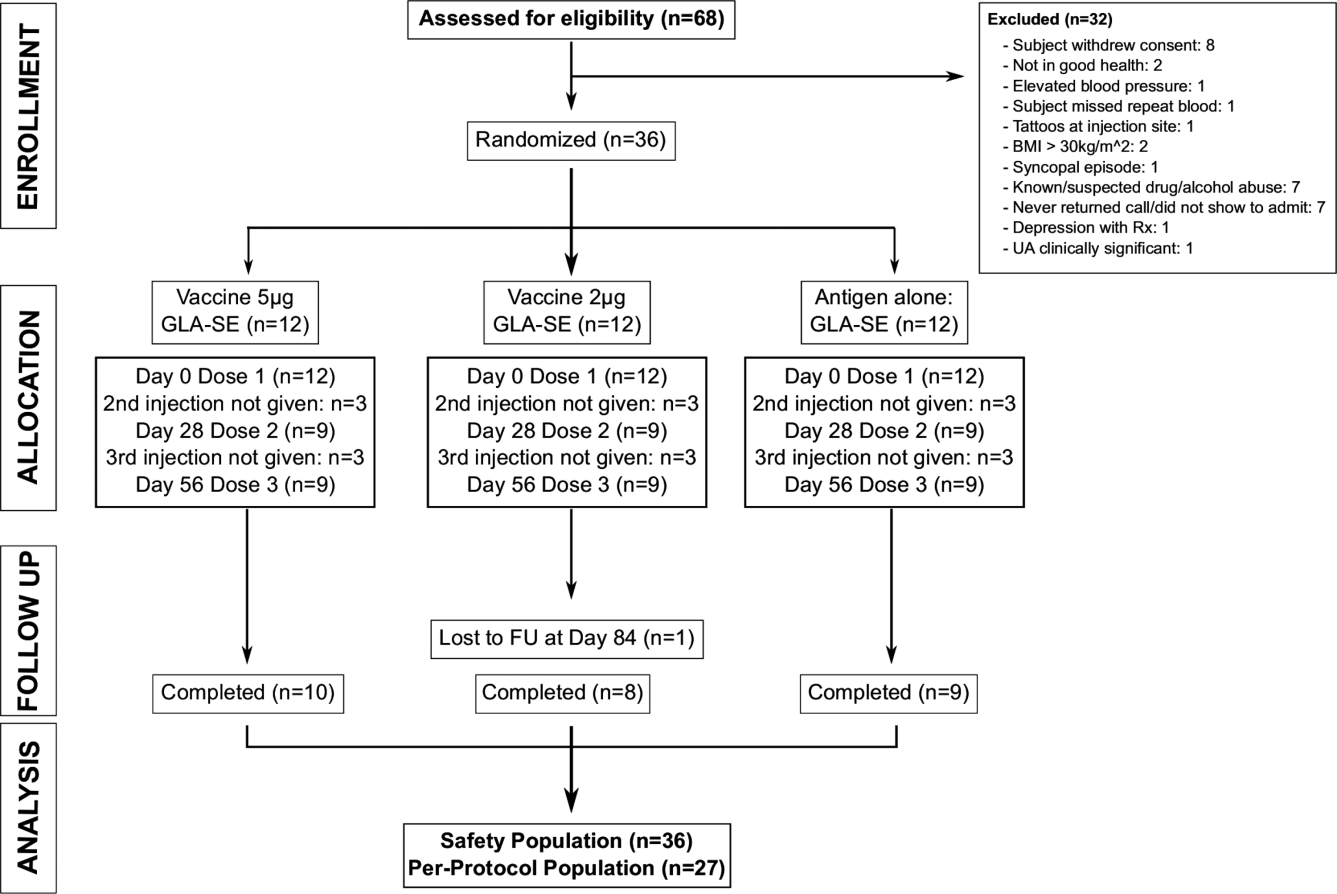
Clinical trial CONSORT diagram. Flow chart shows the number of subjects entering the study from enrollment, allocation and follow-up (FU). Subjects missing the second and third injection dose either declined to continue or were lost to follow-up. Subjects were in follow-up for 1 year after the third injection. The safety population is defined as all subjects who received at least one study injection; the per-protocol population comprises subjects who received all three study injections and completed day 84.

Having demonstrated protection against the causative strains of VL, *L. donovani* and *L. infantum*, with prophylactic immunization in preclinical mouse models, the LEISH-F3+GLA-SE vaccine was advanced to clinical trials to evaluate the safety, tolerability and immunogenicity of the vaccine. Thirty-six volunteers were randomized, enrolled and received at least one study injection; 12 subjects received the vaccine 20 μg LEISH-F3+2 μg GLA-SE, 12 subjects received the vaccine 20 μg LEISH-F3+5 μg GLA-SE and 12 received 20 μg LEISH-F3 protein alone (see Supplementary Table 1). Each volunteer received three intramuscular injections, administered at 28 -day intervals. Eight of 12 subjects in the 20 μg LEISH-F3+2 μg GLA-SE group, 10 of 12 in the 20 μg LEISH-F3+5 μg GLA-SE group and 9 of 12 subjects in the 20 μg LEISH-F3-alone group completed the final day 421 visit. The remainders withdrew consent or were lost to follow-up before completing the study (Figure 5).

#### Safety evaluation

The vaccine was safe and well tolerated in healthy adults, with no serious adverse events (AEs), no AE of special interest and no AE of grade 3 or grade 4 toxicity observed in any of the study groups. The most frequent local reactogenicity AE was tenderness/pain, which occurred in 95.8% of vaccine recipients and 41.7% of antigen-only recipients (Table 2a). The most frequent systemic reactogenicity AE was fatigue, which occurred in +50.0% of vaccine recipients and 33.3% of antigen-only recipients (Table 2b). The most common AE were injection site tenderness/pain, fatigue and decreased hemoglobin (Table 2c). Injection site tenderness/pain was the only AE found to have a statistically significant difference in frequency between the groups that received the vaccine and antigen alone (*P*<0.001). There were no clinically significant changes in clinical chemistry or hematology values. These data indicate that the vaccine was safe and well tolerated at either dose level.

#### Vaccine-induced immune responses

**Figure 6:**
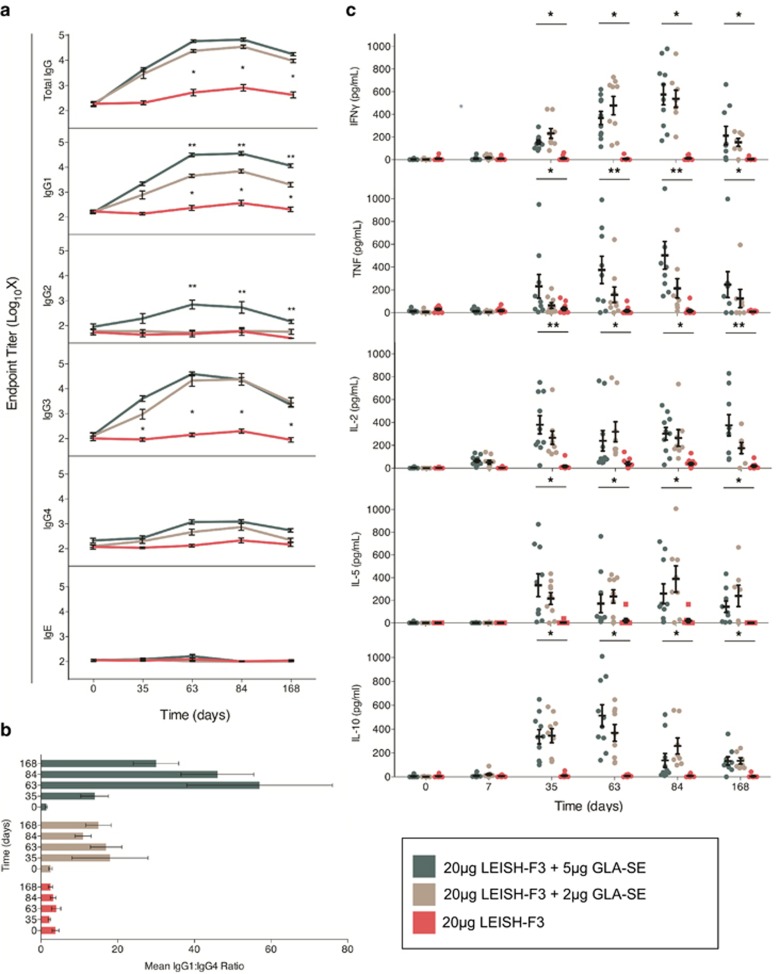
LEISH-F3 formulated with GLA-SE is immunogenic in humans. Thirty-six healthy adult subjects were immunized on days 0, 28 and 56 with 20 μg LEISH-F3+2 μg GLA-SE (*n*=12), 20 μg LEISH-F3+5 μg GLA-SE (*n*=12) and 20μg LEISH-F3 protein alone (*n*=12). The immunogenicity of the vaccine was evaluated by assessing antibody and T-cell responses at days 0, 35, 63, 84 and 168 and days 0, 7, 35, 63, 84 and 168, respectively. (**a** and **b**) ELISAs for titers of LEISH-F3-specific antibodies (total and IgG subclasses and total IgE) in volunteer serum were conducted for the indicated time points. (**c**) Quantitative T-cell responses to LEISH-F3 was measured by IL-2, IL-5, IL-10, IFN-γ and TNF cytokine production in whole blood Luminex assay (WBA). Nine subjects were excluded from the per-protocol population immunology summaries resulting in evaluable groups such as: 2 μg GLA-SE (*n*=8), 5 μg GLA-SE (*n*=10) and LEISH-F3 alone (*n*=9). *P*-value for comparison was performed for various treatment groups: between 2 and 5 μg GLA-SE vaccine groups and between vaccine (2 and 5 μg GLA-SE vaccine groups combined) and 20 μg LEISH-F3 alone. *P*-values were considered significant at the 0.05 significance level. *P-values significant at 0.05 significance level when compared between vaccine (2 and 5 μg GLA-SE vaccine groups combined) and 20 μg LEISH-F3 alone. **P-values significant at 0.05 significance level when compared between vaccine (2 and 5 μg GLA-SE vaccine groups separately).

The antigen-specific immune responses of volunteers who received all three study injections were evaluated. In terms of both the humoral and cellular responses to LEISH-F3 (Figure 6 and Supplementary Figure S2), the vaccine was immunogenic at both 2 and 5 μg of GLA-SE doses. Although subjects vaccinated with the LEISH-F3 protein alone did not develop antigen-specific antibody responses, subjects vaccinated with LEISH-F3+GLA-SE had significant levels of antigen-specific IgG antibodies in their serum. These elevations were evident at day 35 of observation and were maintained up to the last study time point of day 168 (Figures 6a and b). An assessment of IgG subclasses revealed a preferential increase in IgG1 and IgG3 subclasses in the GLA-SE-containing groups (Figure 6a). Antigen-specific IgE was not detected at any time nor in any group (Figure 6a). As the induction of IgG1 is IFNγ-dependent and IgG4 is dependent on IL-4, a preferential induction of isotypes supported by T_H_1-like cytokines, is indicative of a T_H_1 response driven by the addition of GLA-SE.^41, 42^

Whole blood assay (WBA) of subjects injected with protein alone did not demonstrate any antigen-specific cytokine responses at any time point. However, at day 35, the secretion of IFNγ, TNF and IL-2 in response to antigen was observed by WBA of subjects injected with LEISH-F3+GLA-SE. Secretion of these cytokines was further elevated at days 63 and 84, indicating that additional immunizations boosted the cellular response (Figure 6c). Responses were still detected at day 168, although they were lower than those measured at day 84 for IFNγ and TNF, indicating a slight waning of the response over time. IL-2 cytokine responses alternatively remained consistently high through the last time point tested at day 168. Unlike observations in immunized mice, secretion of IL-5 and IL-10 was observed by WBA of subjects injected with LEISH-F3+GLA-SE (Figure 6c). However, IL-10 responses showed a marked decrease after the peak response following day 63. Additionally, in the GLA-SE-containing groups, a significant difference was observed in the group that received 5 μg GLA-SE in terms of increased TNF production at days 63 and 84 and day 168 for IL-2 when compared with the 2 μg GLA-SE group. Furthermore, we evaluated the cytokine responses of PBMC from LEISH-F3-vaccinated subjects when recalled with the LEISH-F3 components (Supplementary Figure S2). As expected, subjects immunized with LEISH-F3+GLA-SE had significant levels of antigen-specific IgG antibodies to both the NH and SMT components compared with the antigen-alone group. No differences were observed in the antibody responses to the components between the 2 and 5 μg adjuvant dose groups of GLA-SE. The cytokine responses to the components reflected the pattern seen with the fusion protein LEISH-F3. An increased T_H_1 and T_H_2 cytokine response profile to the LEISH-F3 components were seen for the adjuvanted groups compared with antigen alone at day 63, and this was further characterized at day 168 by a decrease in IL-10 and IL-5 and a persistence of IL-2 (against NH and SMT) and TNF (against NH) responses (Supplementary Figure S2). Taken together, these data indicate that LEISH-F3+GLA-SE vaccination is safe in humans and induces antigen-specific cellular responses that could protect against *Leishmania* infection.

## DISCUSSION

Strategic framework to eliminate VL in South-East Asia by 2015 has reported a decline in incidence of ~35% owing to implementation of single dose AmBisome and indoor residual spraying of insecticides. Coordinated efforts to improve active case searches as well as early detection and diagnostics services have also reported declining incidences in East Africa (http://www.unitingtocombatNTDs.org). Nevertheless, resistance to affordable first-line treatment options such as pentavalent antimonials has developed, and vector control efforts on disease incidence and transmission have yielded varied results.^4, 5, 43, 44, 45^ Control of leishmaniasis could be achieved by developing new vaccines or less toxic drugs. Regardless of the strategies adopted for elimination, we believe that, based on the intransigent nature of VL, it is important to develop tools to ensure that elimination is complete. To date, no disease has been eliminated without an effective vaccine.

We designed the subunit vaccine candidate, LEISH-F3+GLA-SE, to be a cost-effective measure against VL, and performed preclinical analyses of its immunogenicity, safety, toxicity and efficacy against challenge with *L. donovani* and *L. infantum*. LEISH-F3 consists of the fusion of two antigens each of which were recognized by humans and were previously shown to confer partial protection in mouse models.^16, 17, 19^ LEISH-F3+GLA-SE immunization induced antigen-specific antibody and T-cell immune responses in mice and humans. Induction of predominantly T_H_1-type CD4 T-cell responses was associated with reduced parasite burdens in the livers of vaccinated mice. Furthermore, we demonstrated that immunization with LEISH-F3+GLA-SE in mouse models was protective against both of the causative species of VL, *L. donovani* and *L. infantum.*

A considerable number of *Leishmania* antigens including TSA, LmSTI1, A2, LeIF, Leish-111f, KMP-11, KSAC, CPB, HbR, H2A/H2B, p36/LACK and ORFF have been tested as subunit protein or nucleic acid vaccine candidates against VL and have had variable or unreproducible success in inducing protection.^35, 46, 47, 48, 49, 50, 51, 52, 53, 54, 55, 56, 57, 58, 59^ As demonstrated for both leishmaniasis and tuberculosis, combining multiple antigens in recombinant fusion proteins such as LEISH-F2/LEISH-111f, Mtb72f, ID93, H56, Ag85B-ESAT6, ESAT6-TB10.4, CSU-F36 and Ag85B-TB10 leads to increased vaccine efficacy.^57, 58, 60, 61, 62, 63, 64, 65, 66, 67^ Of these, the multicomponent vaccine LEISH-F2/LEISH-111f+MPL-SE was the first defined vaccine candidate to progress to human clinical trials in healthy volunteers in CL and ML patients in Brazil and Peru and healthy subjects in India.^50, 68, 69, 70, 71^ Because of their low intrinsic immunogenicity, protein-based vaccines need suitable adjuvant systems for the induction of strong *in vivo* immune responses. Several of the most advanced subunit vaccine candidates against non-viral disease targets currently in human clinical trials, RTS, S (malaria vaccine^72^), ID93, Mtb72F, H56 (TB vaccine^23, 65, 73^), are based on liposomal (AS01, CAF01) or oil-in-water (GLA-SE, MPL-SE) formulations, some of which contain the TLR-4 agonist GLA or MPL. Our selection of GLA-SE was based on its ability to activate dendritic cells in a TLR-4-dependent manner and potentiate T_H_1-type CD4 T-cell responses,^22, 26, 61^ which are critical for controlling intracellular infections.

Three doses of the LEISH-F3+GLA-SE vaccine induced antigen-specific T_H_1 responses in mice and humans. Increased production of IFNγ:IL-5 ratios were observed in BALB/c mice. IFNγ, TNF and IL-2 are involved in protection against VL^39, 74, 75, 76^ and TNF has been shown to synergize with IFNγ to kill *Leishmania* parasites.^77^ Therefore, induction of LEISH-F3-specific T cells capable of producing multiple cytokines upon antigen recall might be beneficial for control of *Leishmania* infection, and such induction may be a good indicator of whether a vaccine will be protective against leishmaniasis. In mice, the ability of LEISH-F3+GLA-SE to induce polyfunctional T-cell responses was associated with a statistically significant reduction in liver parasite burden after infection with *L. donovani*, in both BALB/c and C57BL/6 mouse models, or *L. infantum* in BALB/c mice.

Given that the two antigens comprising LEISH-F3 each provide robust protection against challenge with multiple *Leishmania* species,^15, 16, 17, 18, 19, 20^ lack homology with mammalian proteins and are conserved among multiple Old and New World species, we suggest that LEISH-F3 may prove to be a superior vaccine candidate for the spectrum of leishmaniasis diseases. The LEISH-F3-specific cytokine response in vaccinated clinical trial subjects was a complex mixture of T_H_1- and T_H_2-type responses, a phenotype consistent with observed T-cell memory responses in successful vaccine regimens, such as for yellow fever.^78^ Without a definitive correlate of protective immunity against leishmaniasis, vaccine development has had to rely on screening a range of cytokines to estimate the balance between T_H_1 and T_H_2 responses and this balance has been shown to be involved in the outcome of human leishmaniasis.^79^ We showed that, in addition to its T_H_1 response-inducing activity, LEISH-F3 induces IL-10 in vaccinated individuals. Although a balanced response that includes IL-10 may prevent the development of pathology associated with an excessively robust proinflammatory immune response, the induction of IL-10 is a potential concern that will be monitored in future leishmaniasis clinical trials.^79^ In conclusion, these results support the advancement of the LEISH-F3+GLA-SE leishmaniasis vaccine and plans for additional phase 1 clinical trials in endemic countries are underway.

## METHODS

### Recombinant proteins

Recombinant proteins were cloned and expressed in *E. coli* as described previously.^58, 80, 81^ The LEISH-F3 fusion protein was constructed by aligning the individual gene sequences for NH and SMT as a single product. Proteins were quantified using the BCA protein assay (Pierce, Rockford, IL, USA) and were all <100 endotoxin units per mg protein as measured by Limulus Amebocyte Lysate QCL-1000 assay (Lonza Inc., Basel, Switzerland). LEISH-F3 (2 μg per lane) was analyzed by reducing and nonreducing sodium dodecyl sulfate-polyacrylamide gel electrophoresis (SDS-PAGE). LEISH-F3, NH, SMT (100 ng per lane) or *L. donovani* whole-cell lysate (27 μg per lane) were immunoblotted with rabbit polyclonal antibody to LEISH-F3 and with mouse monoclonal antibody against NH or SMT. To demonstrate the absence of host cell proteins, LEISH-F3 (2 μg per lane) was immunoblotted with rabbit polyclonal antibody to HMS174 *E. coli*, with HMS174 *E. coli* lysate as a positive control.

### Recognition of NH and SMT in samples from individuals from a *Leishmania*-endemic area

Sera were obtained from residents of a *Leishmania*-endemic area of Bangladesh (VL patients and asymptomatics) or the United States (non-endemic normal). Written informed consent for study participation was obtained from each participant before screening. Subjects were defined according to parameters set by the Kala-Azar Elimination Program as follows: VL, a person with clinical symptoms of VL (fever for more than 2 weeks duration and splenomegaly) and a positive rK39 RDT result; asymptomatic individual, a person from an endemic area with no present or past clinical symptoms of VL but with a DAT titer >600; non-endemic, healthy individuals living out with endemic regions reporting no history of VL. ELISA was conducted as previously described with the sera at a 1:400 dilution.^82^

*Intracellular cytokine staining*: PBMCs were isolated from peripheral blood of the subjects following the standard Ficoll procedure. PBMCs were stimulated with NH and SMT, phosphate-buffered saline, with costimulatory antibodies CD28 and CD49d (BD Biosciences, San Jose, CA, USA), in complete RPMI at 37 °C. Cells were then stained for membrane expression of CD3 (clone UCHT1; Beckman Coulter, Pasadena, CA, USA), CD4 (OKT4) and CD8 (HIT8a) (BD Bioscience). Cells were fixed and permeabilized in Cytofix/Cytoperm (BD Biosciences) according to the manufacturer's instructions and stained for intracellular expression of IFN-γ (4S.B3), TNF (MAb11) and IL-2 (MQ1-17H12) (BD Bioscience). Events were acquired using a BD LSRFortessa (BD Biosciences) and the data were analyzed with FlowJo (Tree Star Inc., Ashland, OR, USA). For analyses, the cells were first gated at CD3 to identify CD4 and CD8 T cells, and then gated for IFNγ, TNF and IL-2. Responses are shown as background (phosphate-buffered saline)-subtracted percent antigen-specific T cells producing the indicated cytokines.

### Mouse treatments

All animal procedures were approved by the IDRI institutional animal care and use committee. Female C57BL/6 and BALB/c mice were purchased from Charles River Laboratories (Wilmington, MA, USA). Mice were maintained in specific pathogen-free conditions in the animal facilities of the IDRI and entered experiments at 6–8 weeks of age. Mice were immunized three times at 3-week intervals by injection at the base of the tail. Immunizations were made in a volume of 100 μl per dose as follows: 5 μg per dose of LEISH-F3; 5 μg of GLA in stable nanoemulsion (SE) of 2% oil (GLA-SE).

CD4 or CD8 T cells were depleted by intraperitoneal injection of 0.5 mg of either CD4 (clone GK1.5), CD8 (clone 53-6.72) or a rat IgG2a isotype control (Bio X Cell, West Lebanon, NH, USA) on 3 consecutive days. Depletion was confirmed by flow cytometry analysis of blood. Three weeks after depletion, mice were infected with *L. donovani*.

### Antibody analyses

Antigen-specific serum antibodies were measured by ELISA. End-point titers were calculated using the optical density >0.1 as a cutoff value with GraphPad Prism 6 (GraphPad Software Inc., La Jolla, CA, USA). End-point titers of samples were recorded as <100 if optical density values of the samples were lower than the cutoff value at 1:100 dilution.

### Cell preparations and antigen stimulation assays

Four weeks after the final immunization, spleens were removed and single-cell suspensions were prepared. Splenocytes were enumerated using a ViaCount assay with a PCA system (Guava Technologies, Darmstadt, Germany), and then incubated with media only or 10 μg ml^−1^ antigen.

*ELISPOT assays:* IFNγ and IL-5 ELISPOTs (R&D Systems, Minneapolis, MN, USA) were conducted according to the manufacturer's instructions. Spot images were collected using ImmunoCapture 6.4 and analyzed with ImmunoSpot 5.0 on an automated ELISPOT plate reader (C.T.L. Seri3A Analyzer; Cellular Technology, Shaker Heights, OH, USA).

#### Secreted cytokines detection

Culture supernatants were collected after 72 h, and IFNγ, TNF, IL-2, IL-5 and IL-10 were determined using bead-based multiplex assay with BD Biosciences' cytometric bead array (CBA) flex set according to the manufacturer's instructions (BD Biosciences).

#### Intracellular cytokine staining and flow cytometry

Splenocytes were cultured with antigen plus 1 μg ml^−1^ each of the costimulatory antibodies anti-mouse CD28 and CD49d (BD Biosciences), and then stained for membrane expression of CD90.2 (30-H12), CD4 (GK1.5) and CD8 (53-6.7 (BD Biosciences). Cells were then fixed and permeabilized in Cytofix/Cytoperm (BD Biosciences) according to the manufacturer's instructions and stained for intracellular expression of CD154 (MR1; eBioscience), IFNγ (XMG1.2), TNF (MP6-XT22) and IL-2 (JES6-5H4) (BD Bioscience). Events were acquired using a BD LSRFortessa (BD Biosciences) and the data were analyzed with FlowJo (Tree Star Inc.), Pestle and SPICE (NIAID, NIH (BCBB, Bethesda, MD, USA)). Cells were gated through CD90.2^+^ T cells to analyze CD4 and CD8 T cells. Intracellular molecules were analyzed after gating through CD4^+^CD8^−^ T cells. For CD4 epitope mapping analysis, cells were gated and analyzed for CD154 expression.

#### Infection and parasite quantification

*L. donovani* (MHOM/SD/00/1S-2D) and *L. infantum* (MHOM/BR/82/BA-2) were routinely passed through Syrian golden hamsters to generate virulent amastigote and promastigote stocks in the M199 medium. Mice were challenged by retro-orbital intravenous injection with either 1 × 10^6^  L*. donovani* or 5 × 10^6^  L*. infantum* parasites. Three or four weeks after infection, livers were harvested and DNA was extracted from homogenate using QIAmp DNA Mini Kits (Qiagen, Hilden, Germany). DNA was quantified using Nanodrop UV-Vis spectrophotometer (ND-1000). *L. donovani* DNA was detected using primers for L42486 (forward, 5′-GCGACGTCCGTGGAAAGAA-3′ and reverse, 5′-GGCGGGTACACATTAGCAGAA-3′) with a FAM reporter sequence (5′- CAACGCGTATTCCC-3′), which detects a 203- bp genomic repeat region specific to *Leishmania* species (NCBI Blastn). *L. infantum* primers used were described previously.^83^ Mouse Gapdh FAM (Life Technologies, Carlsbad, CA, USA) was used as an internal reference control. Cp's of samples were fitted to a standard curve to determine number of parasite per μl of DNA. Final parasite burdens are expressed in the number of *Leishmania* parasites per organ.

### Statistical methods

Data for human ELISA were compared by analyses of non-endemic normals vs VL patients or asymptomatics. Data for intracellular cytokine staining were compared by analyses of non-endemic vs asymptomatics. Statistics were calculated by a Kolmogorov–Smirnov test using GraphPad Prism Version 6.01 (GraphPad Software, San Diego, CA, USA).

For data generated using mouse samples, statistics was determined by one- or two-way ANOVA (GraphPad Prism). Significance was considered when the *P*-values were <0.05.

### Clinical methods

*Trial design:* The safety, tolerability and immunogenicity of LEISH-F3+GLA-SE was evaluated in a first-in-man phase 1, randomized, dose-escalation study. A total of 36 healthy adults (male and female adults with no history of travel to *Leishmania*-endemic areas) were recruited after signing the informed consent form, and then randomly assigned to receive injections with 20 μg LEISH-F3 in combination with either nothing, 2 μg GLA-SE or 5 μg GLA-SE (12 per group) at 28-day intervals (days 0, 28 and 56). The treatment randomization list was generated using SAS software (SAS Institute Inc., Cary, NC, USA). The study biostatistician filled a set of individual, opaque, sealed envelopes, each labeled with a unique subject/participant identification number and containing the corresponding treatment assignment. Only the designated pharmacist(s) responsible for study injection preparation had access to the randomization assignment envelopes. All study injections were 0.5 ml in volume and were prepared no more than 2 h before administration. The investigators who evaluated study subjects for reactogenicity and AEs were blinded to study treatment assignments (only the study pharmacists were unblinded).

Twenty-four subjects were granted Medical Monitor approval for laboratory eligibility criteria deemed not clinically significant. In addition, the day 421 follow-up telephone visit for subjects was performed ~4 months early due to closure of the study site.

Out of 36 subjects who were randomized to injection, seven subjects withdrew consent or were lost to follow-up before receiving all three injections (three in the 2 μg GLA-SE group, two in the 5 μg GLA-SE group and two in the LEISH-F3-alone group), one subject (in the LEISH-F3 alone group) missed the day 56 evaluation and did not receive a third injection, and one subject (in the 2 μg GLA-SE group) was lost to follow-up before day 84. Thus, nine subjects were excluded from the per-protocol population immunology summaries resulting in evaluable groups for the ELISA (against LEISH-F3, NH and SMT) and WBA (against LEISH-F3) as follows: 2 μg GLA-SE (*n*=8), 5 μg GLA-SE (*n*=10) and LEISH-F3 alone (*n*=9). Final resulting evaluable groups for PBMC luminex (against NH and SMT) were: 2 μg GLA-SE (*n*=6), 5 μg GLA-SE (*n*=9) and LEISH-F3 alone (*n*=7).

#### Safety evaluations

The primary safety end points were the proportion of subjects with AEs, serious AEs, AEs of special interest, local injection site reactions and specific systemic reactions to the study injections from days 0 to 84. The occurrence of AEs, SAEs and AEs of special interest was monitored from the time of the first study injection at day 0 through day 421, and the investigator assessed their relation to study injections and severity based on the Food and Drug Administration Guidance for Industry Toxicity Grading Scale for Healthy Adults Enrolled in Preventive Vaccine Clinical Trials, 2007.^84^ AE grades for laboratory parameters were set based on normal laboratory values. For non-laboratory conditions, AEs that were mild and did not limit activity were considered grade 1; those that were moderate and interfered with function, but not with activities of daily living, grade 2; those that were severe and interfered with activities of daily living, grade 3; and those that were life-threatening or disabling, grade 4. Serious AEs were those that resulted in death, were considered life-threatening, required hospitalization or prolongation of hospitalization, or resulted in persistent or significant disability, or resulted in a congenital anomaly or birth defect in the offspring of a study patient. AEs of special interest were those that met the Food and Drug Administration list for specific AEs. The occurrence of local injection site reactions was assessed at 30 min, 2 days and 7 days following each study injection. The occurrence of the specific systemic reactions headache, anorexia, malaise/fatigue, muscle pain, fever, hives, rash and chills was evaluated in the 7 days following each study injection. Blood samples were collected for measurement of hematology and serum chemistry parameters at screening and on days 7, 35 and 63. Vital signs were collected at every study visit and before and 30 min after study injection on days 0, 28, and 56.

#### Total IgG antibody assay

High-binding 384-well ELISA plates (Corning, Tewksbury, MA, USA) were coated with recombinant protein, and then serial dilutions of the plasma samples and positive control and a single dilution of negative control were prepared in serum diluent. After incubation, peroxidase-labeled anti-human IgG, IgG1, IgG2, IgG3, IgG4 and IgE (Life Technologies, Carlsbad, CA, USA) in serum diluent was added. End-point titer was determined by Graph Prism Nonlinear regression (curve fit) sigmoidal dose response (variable slope) to interpolate unknowns from the last optical density value greater than a threshold determined by sera from normal human plasma pool.

#### Antigen-specific cell stimulations

Cells or blood were incubated with recombinant protein (10 μg ml^−1^), phosphate-buffered saline or a positive control mitogen (phytohemaglutinin, 10 μg ml^−1^). PBMCs were isolated from peripheral blood of the vaccinated subjects following the standard Ficoll procedure. Alternatively, for whole blood assay, unfractionated peripheral blood from vaccinated subjects was incubated within 2 h of collection. Cytokine (IL-2, IL-5, IL-10, IFNγ and TNF) concentrations in the supernatants were measured with a Milliplex Multiplexed Bead Array Kit (EMD Millipore, Billerica, MA, USA) as per the manufacturer's instructions, with the exception that a nine-point standard curve was applied to provide higher statistical power for curve-fitting regressions (Millipore, Billerica, MA, USA). Data were collected and analyzed on Luminex 200 instrument (Millipore) using Masterplex software (MiraiBio, South San Francisco, CA, USA).

## Figures and Tables

**Table 1:** Homology of LEISH-F3 antigens, NH and SMT, among *Leishmania* species

*Leishmania species*	*Percent homology*	
	*NH*	*SMT*
*L. donovani*	100	99
*L. infantum*	99	100
*L. major*	96	97
*L. tropica*	98	NA
*L. mexicana*	93	94
*L. braziliensis*	84	86
*L. guyanensis*	84	86
*L. amazonensis*	NA	94
*L. aethiopica*^a^	30	NA

Abbreviations: NA, not available; NH, nucleoside hydrolase; SMT, sterol 24-c-methyltransferase.

a Alignments for partial due to NA of complete sequences

**Table 2:** Safety evaluations for the phase 1 clinical trial

*AE*	*LEISH-F3*		
	*20 μg*	*20 μg*	*20 μg*
	*GLA-SE*		
	*2 μg*	*5 μg*	*—*
	(n=*12)*	(n=*12)*	(n=*12)*
*(A)*			
Any local reaction	11 (91.7%)	12 (100%)	6 (50.0%)
Postinjection period 1	10 (83.3%)	12 (100%)	5 (41.7%)
Postinjection period 2	7 (58.3%)	9 (75.0%)	3 (25.0%)
Postinjection period 3	4 (33.3%)	7 (58.3%)	1 (8.3%)
Injection site tenderness/pain	11 (91.7%)	12 (100%)	5 (41.7%)
Injection site induration/swelling	3 (25.0%)	1 (8.3%)	1 (8.3%)
Injection site erythema/redness	3 (25.0%)	0 (0%)	0 (0%)
Injection site ecchymosis	1 (8.3%)	0 (0%)	2 (16.7%)
			
*(B)*			
Any systemic reaction	5 (41.7%)	7 (58.3%)	5 (41.7%)
Postinjection period 1	4 (33.3%)	4 (33.3%)	4 (33.3%)
Postinjection period 2	1 (8.3%)	5 (41.7%)	1 (8.3%)
Postinjection period 3	1 (8.3%)	1 (8.3%)	0 (0%)
Anorexia	3 (25.0%)	2 (16.7%)	1 (8.3%)
Chills	0 (0%)	1 (8.3%)	0 (0%)
Fatigue	5 (41.7%)	7 (58.3%)	4 (33.3%)
Headache	1 (8.3%)	1 (8.3%)	0 (0%)
Myalgia	1 (8.3%)	2 (16.7%)	0 (0%)
			
*(C)*			
Injection-related reactions			
Anorexia	3 (25.0%)	2 (16.7%)	1 (8.3%)
Fatigue	5 (41.7%)	7 (58.3%)	4 (33.3%)
Injection site induration/swelling	3 (25.0%)	1 (8.3%)	1 (8.3%)
Injection site tenderness/pain	11 (91.7%)	12 (100%)	5 (41.7%)
Laboratory Investigations			
Hemoglobin decreased	2 (16.7%)	4 (33.3%)	5 (41.7%)
White blood cell count decreased	2 (16.7%)	1 (8.3%)	3 (25.0%)
General AEs			
No event occurring in ⩾5 subjects			

Abbreviations: AE, adverse event; GLA-SE, glucopyranosyl lipid A-stable oil-in-water nanoemulsion.

(A) Frequency of local reactogenicity AEs in all subjects. Postinjection periods were defined as period 1: days 0–7; period 2: days 28–35; and period 3: days 56–63. (B) Frequency of systemic reactogenicity AEs in all subjects. (C) Proportion of subjects with AEs occurring in 5 or more subjects in study period 1. The occurrence of local injection site reactions was assessed at 30 min, 2 days and 7 days following each study injection. The occurrence of the specific systemic reactions headache, anorexia, malaise/fatigue, muscle pain, fever, hives, rash and chills was evaluated in the 7 days following each study injection. Blood samples were collected for measurement of hematology and serum chemistry parameters at screening and on days 7, 35 and 63. Vital signs were collected at every study visit and before and 30 min after study injection on days 0, 28 and 56.

## Notes

SR is a coinventor on a patent for leishmaniasis vaccine development. All the other authors declare no conflict of interest.
