# Supplementary material for: From mouse to man: safety, immunogenicity and efficacy of a candidate leishmaniasis vaccine LEISH-F3+GLA-SE
Source: Clin Transl Immunology. 2015 Apr 10;4(4):e35–. doi: 10.1038/cti.2015.6 (PMC4488838; doi:10.1038/cti.2015.6)
Supplement: Supplementary Table 1 [file cti20156x1.doc]

Supplemental Table 1: Summary of Demographic Data and Other Baseline

|  | **20 µg LEISH-F3 + 2 µg GLA-SE (n=12)** | **20 µg LEISH-F3 + 5 µg GLA-SE (n=12)** | **20 µg LEISH-F3 (n=12)** | **P-value^1^** | **P-value^2^** | **P-value^3^** |
| --- | --- | --- | --- | --- | --- | --- |
| **Age** |  |  |  |  |  |  |
| **Mean** | 26.1 | 26.6 | 30.4 | 0.321 | 0.859 | 0.177 |
| **Std Deviation** | 6.22 | 7.40 | 8.87 |  | | |
| **Std Error** | 1.79 | 2.14 | 2.56 |
| **Median** | 26.0 | 24.0 | 33.0 |
| **Min - Max** | 19 - 40 | 18 - 40 | 18 - 42 |
| **Gender** |  |  |  |  | | |
| **Female** | 6 ( 50.0%) | 7 ( 58.3%) | 7 ( 58.3%) | 1.000 | 1.000 | 1.000 |
| **Male** | 6 ( 50.0%) | 5 ( 41.7%) | 5 ( 41.7%) |  | | |
| **Race** |  |  |  |  | | |
| **White** | 5 ( 41.7%) | 7 ( 58.3%) | 9 ( 75.0%) | 0.218 | 0.904 | 0.097 |
| **Asian** | 0 | 0 | 2 ( 16.7%) |  | | |
| **Black/African American** | 4 ( 33.3%) | 4 ( 33.3%) | 1 ( 8.3%) |
| **Hawaiian/Pacific Islander** | 1 ( 8.3%) | 0 | 0 |
| **Mixed Race** | 1 ( 8.3%) | 1 ( 8.3%) | 0 |
| **Other: Taino** | 1 ( 8.3%) | 0 | 0 |
| **BMI (kg/m^2^)** |  |  |  |  | | |
| **Mean** | 23.0 | 24.7 | 26.2 | 0.047* | 0.166 | 0.050* |
| **Std Deviation** | 2.66 | 3.03 | 3.27 |  | | |
| **Std Error** | 0.77 | 0.87 | 0.94 |
| **Median** | 22.5 | 24.5 | 27.5 |
| **Min - Max** | 19 - 27 | 20 - 29 | 21 - 30 |

^1^ p-value for comparison across all three treatment groups.

^2^ p-value for comparison between 2 µg and 5 µg GLA-SE vaccine groups.

^3^ p-value for comparison between vaccine (2 µg and 5 µg GLA-SE vaccine groups combined) and 20 µg LEISH-F3 alone.

Note: P-values for means obtained by t-tests or ANOVA. P-values for categorical data obtained by Fisher’s exact test. P-values significant at the 0.05 significance level marked with *.
