# Supplementary figures and images for: From mouse to man: safety, immunogenicity and efficacy of a candidate leishmaniasis vaccine LEISH-F3+GLA-SE
Source: Clin Transl Immunology. 2015 Apr 10;4(4):e35–. doi: 10.1038/cti.2015.6 (PMC4488838; doi:10.1038/cti.2015.6)

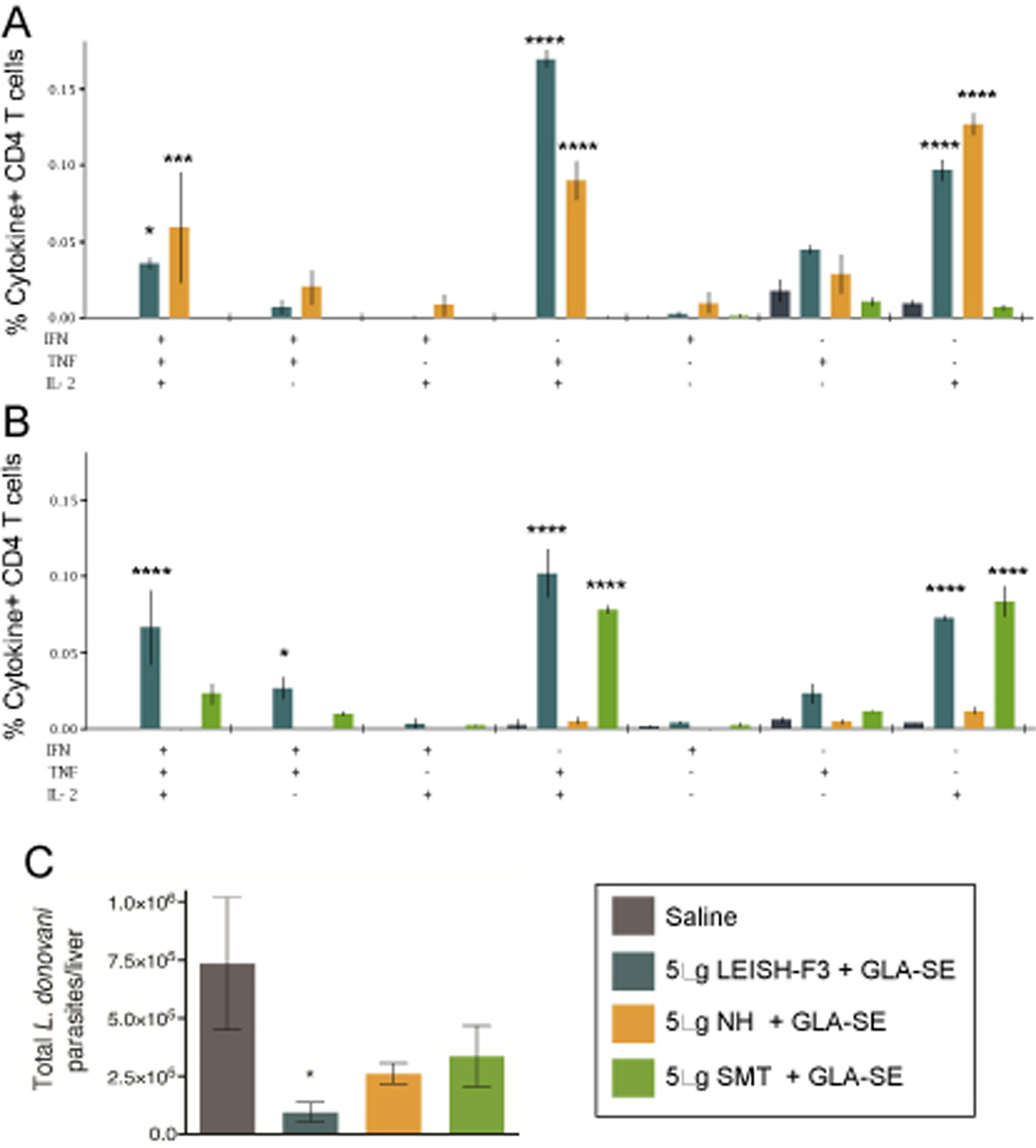

Supplement: Supplementary Figure 1 [file cti20156x2.tif]

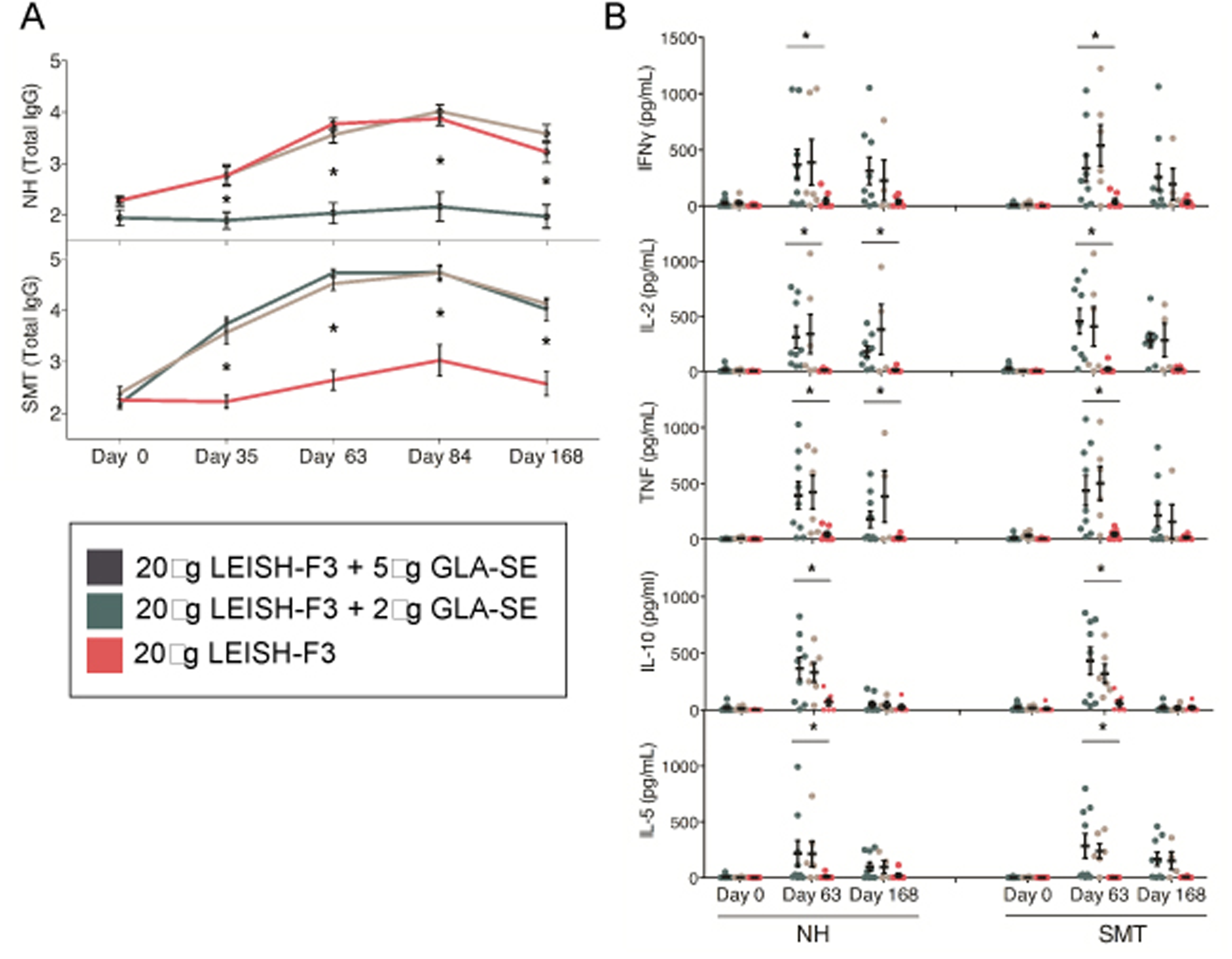

Supplement: Supplementary Figure 2 [file cti20156x3.tif]
