# Supplementary material for: From mouse to man: safety, immunogenicity and efficacy of a candidate leishmaniasis vaccine LEISH-F3+GLA-SE
Source: Clin Transl Immunology. 2015 Apr 10;4(4):e35–. doi: 10.1038/cti.2015.6 (PMC4488838; doi:10.1038/cti.2015.6)
Supplement: Supplementary Information [file cti20156x4.doc]

**Supplemental Figure 1. Immunogenicity and protection of NH/GLA-SE and SMT/GLA-SE.** BALB/c mice were immunized with the indicated formulations three times, at three week intervals. Four weeks after the final immunization, mice were used for (A-B) immunogenicity analysis (n=3) or (C) infected with 1 x 10^6^ *L. donovani* parasites iv (n=7); four weeks after infection liver burdens were determined. (A) NH or (B) SMT-specific immune responses were analyzed by recall of splenocytes with 10μg/mL of protein and intracellular cytokine staining for polyfunctional CD4 T cells expressing combinations of IFNγ, TNF, and IL-2. (C) Liver burdens in infected mice were determined by RT-PCR. Statistics by Dunnett’s multiple comparison test; *p<0.05.

**Supplemental Figure 2. LEISH-F3 immunized human subjects are responsive to LEISH-F3 components NH and SMT.** Healthy adult subjects were immunized on Day 0, 28 and 56 with 20 μg LEISH-F3 + 2 μg GLA-SE (n=12), 20 μg LEISH-F3 + 5 μg GLA-SE (n=12), and 20ug LEISH-F3 protein alone (n=12). The immune responses to LEISH-F3 components of the vaccine was evaluated by assessing antibody and T-cell responses at days 0, 35, 63, 84 and 168 and days 0, 63, and 168 respectively. (A) Total IgG ELISAs for titers of components of LEISH-F3, NH and SMT-specific antibodies in patient serum were conducted for the indicated time points. (B) Quantitative T-cell responses to LEISH-F3 components proteins was measured by IL-2, IL-5, IL-10, IFN-γ and TNF cytokine production in PBMC Luminex assay .

Final resulting evaluable groups were: 2 µg GLA-SE (n=6), 5 µg GLA-SE (n=9), and LEISH-F3 alone (n=7). P-value for comparison was performed for various treatment groups- between 2 µg and 5 µg GLA-SE vaccine groups and between vaccine (2 µg and 5 µg GLA-SE vaccine groups combined) and 20 µg LEISH-F3 alone. P-values were considered significant at the 0.05 significance level.

* p-values significant at 0.05 significance level when compared between vaccine (2 µg and 5 µg GLA-SE vaccine groups combined) and 20 µg LEISH-F3 alone. ** p-values significant at 0.05 significance level when compared between vaccine (2 µg and 5 µg GLA-SE vaccine groups separately).

**Supplemental Table 1: Demographics and clinical characteristics (Baseline Data)**
